# Vitamin D Status in Bipolar Disorder

**DOI:** 10.3390/nu15224752

**Published:** 2023-11-11

**Authors:** Zita Späth, Adelina Tmava-Berisha, Frederike T. Fellendorf, Tatjana Stross, Alexander Maget, Martina Platzer, Susanne A. Bengesser, Alfred Häussl, Ina Zwigl, Armin Birner, Robert Queissner, Katharina Stix, Linda Wels, Melanie Lenger, Nina Dalkner, Sieglinde Zelzer, Markus Herrmann, Eva Z. Reininghaus

**Affiliations:** 1 Clinical Department of Psychiatry and Psychotherapeutic Medicine, Medical University Graz, 8036 Graz, Austria; zita.spaeth@stud.medunigraz.at (Z.S.); frederike.fellendorf@medunigraz.at (F.T.F.); tatjana.stross@medunigraz.at (T.S.); alexander.maget@medunigraz.at (A.M.); martina.platzer@medunigraz.at (M.P.); susanne.bengesser@medunigraz.at (S.A.B.); alfred.haeussl@medunigraz.at (A.H.); ina.zwigl@medunigraz.at (I.Z.); armin.birner@medunigraz.at (A.B.); robert.queissner@medunigraz.at (R.Q.); katharina.stix@medunigraz.at (K.S.); linda.wels@medunigraz.at (L.W.); melanie.lenger@medunigraz.at (M.L.); nina.dalkner@medunigraz.at (N.D.); eva.reininghaus@medunigraz.at (E.Z.R.); 2 Clinical Institute of Medical and Chemical Laboratory Diagnostics, Medical University of Graz, 8036 Graz, Austria; sieglinde.zelzer@medunigraz.at (S.Z.); markus.herrmann@medunigraz.at (M.H.)

**Keywords:** bipolar disorder, vitamin D metabolism, functional vitamin D deficiency, 25(OH)D, 24,25(OH)_2_D, VMR

## Abstract

Vitamin D status may impact acute affective symptomatology and the severity of symptoms in patients with bipolar disorder (BD). Therefore, this cross-sectional study analyzed 25(OH)D, 24,25(OH)_2_D, and the vitamin D metabolite ratio (VMR) in BD and correlated the results with clinical affective symptomatology and functionality. The inactive precursor 25(OH)D, and its principal catabolite 24,25(OH)_2_D, were measured simultaneously with a validated liquid chromatography–tandem mass spectrometry method in 170 BD outpatients and 138 healthy controls. VMR was calculated as follows: VMR = 100×(24,25(OH)_2_D/25(OH)D). The psychometric assessment comprised: Beck Depression Inventory-II, Hamilton Depression Rating Scale, Young Mania Rating Scale, Global Assessment of Functioning, and number of suicide attempts. We did not find a significant difference between patients and controls in the concentrations of 25(OH)D and 24,25(OH)_2_D. Additionally, the VMR was comparable in both groups. The calculations for the clinical parameters showed a negative correlation between the Young Mania Rating Scale and 24,25(OH)_2_D (*r* = −0.154, *p* = 0.040), as well as the Young Mania Rating Scale and the VMR (*r* = −0.238, *p* = 0.015). Based on the small effect size and the predominantly euthymic sample, further exploration in individuals with manic symptoms would be needed to confirm this association. In addition, long-term clinical markers and an assessment in different phases of the disease may provide additional insights.

## 1. Introduction

Bipolar disorder (BD) is an affective disorder characterized by tremendous mood swings ranging from depression to mania [1]. During manic episodes, a euphoric mood and/or irritability are accompanied by a decreased need for sleep, enormous energy, overconfidence, and difficulty concentrating. On the contrary, patients in a bipolar depressive episode exhibit a depressed mood, reduced energy, anhedonia, reduced libido, and an elevated risk of suicide [2]. The range of lifetime prevalence for bipolar type I disorder is 0.3–1.5% and up to 5.5% for bipolar type II disorder with high heritability [3].

Subjects with BD have a high prevalence of somatic comorbidities (such as hypertension, diabetes mellitus, etc.) due to an unhealthy lifestyle, the side effects of medication, and neuroinflammatory processes including oxidative stress [4,5,6,7,8]. Many patients also exhibit cognitive deficits associated with low everyday functioning [3]. Additionally, the illness’s course can be influenced by lifestyle and nutrition [9]. Furthermore, Bauer et al. (2014) described an inverse association between the age at onset of BD and the magnitude of annual variation of sunlight exposure; namely, the larger the variation of sunlight throughout the year, the earlier the onset of BD. Sunlight is not only important for the circadian rhythm and thermal effects, but also plays a major role in the production of vitamin D [10].

Vitamin D is a fat-soluble vitamin and has been shown to be associated with a multitude of somatic functions, but also psychological well-being [6,11,12,13]. Vitamin D metabolism is complex and involves multiple hydroxylation steps, resulting in the production of the active form of vitamin D (1,25(OH)_2_D), which has endocrine, paracrine, and autocrine effects [14,15,16].

Thus far, vitamin D status has been assessed by measuring the inactive precursor 25(OH)D, which provides information on vitamin D stores but not on functional aspects. Furthermore, the measurement of 25(OH)D through widely available immunoassays is lacking accuracy, which hampers comparability between labs. Liquid chromatography– tandem mass spectrometry (LC-MS/MS) allows the simultaneous determination of 25(OH)D and its main catabolite 24,25(OH)_2_D with high sensitivity and accuracy, providing additional metabolic information. Detectable amounts of 24,25(OH)_2_D imply that vitamin D stores are sufficient to maintain adequate vitamin D metabolism, as there is no 25(OH)D to spare for catabolism. In contrast, undetectable 24,25(OH)_2_D concentrations suggest a functional vitamin D deficiency [17,18]. Furthermore, the vitamin D metabolite ratio (VMR) aids in the dynamic assessment of vitamin D metabolism status [18,19].

Vitamin D has neuroprotective and proliferative effects on brain cells. Moreover, vitamin D has antioxidant properties via lower cytokine production and ultimately reduces neuroinflammatory processes [13]. Additionally, and very important for its leverage on psychiatric disorders, it is an activator of tyrosine hydroxylase gene expression, an enzyme that presumably catalyzes the rate-limiting step in catecholamine synthesis. Catecholamines, namely, adrenaline, noradrenaline, and dopamine, are linked to mood disorders [13,15]. Furthermore, vitamin D can trespass the blood–brain barrier, and vitamin D receptors can be found in certain parts of the brain—e.g., the cortex, cerebellum, and limbic system –which suggests an influence on the brain [20].

However, the link between vitamin D deficiency and psychiatric diseases is not well-understood, and the current literature is inconsistent [21]. According to Rihal et al. (2022), a deficiency in vitamin D could potentially contribute to the onset of neuropsychiatric disorders, such as schizophrenia, autism, depression, and ADHD [22]. It is already well-accepted that the metabolic actions of vitamin D go far beyond calcium–phosphate homeostasis and involve the brain and many other tissues [23]. Furthermore, alterations in vitamin D levels within neurons have been associated with a decrease in the density of nerve fibers, the delayed and diminished development of dopamine neurons, and impaired release of gamma-aminobutyric acid and glutamate, supporting the role of vitamin D in myelination and the functional recovery of neurons [24]. A recent study showed a possible correlation between low vitamin D values and neurodegeneration, followed by reduced brain volume. This led to the hypothesis that sufficient vitamin D levels could contribute to the preservation of brain health in general [25]. According to existing evidence [21,26,27,28], individuals with BD have an increased risk of vitamin D deficiency, which has adverse effects on sleep and the circadian rhythm [12]. Also, Jorde and Kubiak (2018) considered the possibility of reverse causality, where vitamin D deficiency is a consequence of depression [29]. Furthermore, vitamin D concentrations of individuals with BD and other psychiatric diseases do not differ significantly. While an explanation could be that most psychiatric patients have comparably poor nutrition and spend less time outside, studies on the association between vitamin D levels and the severity of symptoms of BD are nevertheless limited, and none of them included functional assessments by 24,25(OH)_2_D and VMR. 

This study therefore aims to analyze potential differences between individuals with BD and a healthy control group regarding their vitamin D status, including the frequency of functional vitamin D deficiency, and to test for associations between 25(OH)D; 24,25(OH)_2_D; VMR; and clinical characteristics of BD. We hypothesized that (1) individuals with BD have lower 25(OH)D; 24,25(OH)_2_D; and VMR than the healthy controls, (2) the frequency of functional vitamin D deficiency differs between the groups, and (3) vitamin D values (25(OH)D; 24,25(OH)_2_D; VMR) correlate with acute affective symptomatology and functionality in individuals with BD.

## 2. Materials and Methods

### 2.1. Participants

**Figure 1:**
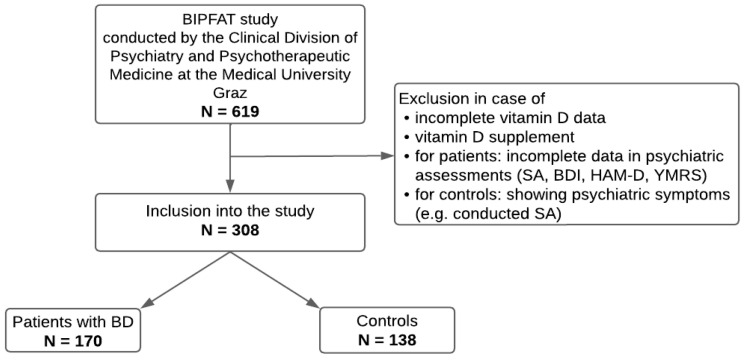
Inclusion process of patients and healthy controls. Note: SA = suicide attempts; BDI = Beck Depression Inventory; HAM-D = Hamilton Depression Rating Scale; YMRS = Young Mania Rating Scale.

This research is part of the ongoing BIPFAT study conducted by the Clinical Division of Psychiatry and Psychotherapeutic Medicine at the Medical University of Graz, Austria. The overall aim of the ongoing study is to uncover possible correlations between BD and cognitive function, genetics, lifestyle, clinical parameters (e.g., vitamin D, cholesterol, inflammatory signs), and somatic comorbidities. The study overlaps with the sample of Leser et al. [30]. The patients included were diagnosed with BD according to the Structured Clinical Interview, according to the DSM-IV criteria, conducted by a psychiatrist or clinical psychologist [2]. We included all patients with bipolar disorder; the distinction between BD I and BD II was not considered in further analysis. Each participant had to have reached the age of 18 and signed a written informed consent. Both in- and outpatients of the dedicated center for BD and healthy control persons took part in this study. Approval by the local ethics committee based on the Declaration of Helsinki was obtained (EK number: 24–123 ex 11/12). For deeper insight and previous results, we hereby refer to former reports (e.g., [31,32,33,34]). A study flow diagram is presented in Figure 1.

### 2.2. Psychometric Assessment

All patients underwent a detailed psychometric assessment, including suicidal attempts, BDI II, HAM-D, YMRS, and GAF. The number of suicidal attempts (SA) was documented through a clinical interview and medical records. Self-reported depressive symptoms were assessed by the German version of the Beck Depression Inventory II (BDI II) [35]), including 21 items. In addition, depressive and manic symptoms were assessed by a psychiatrist or a clinical psychologist using the Hamilton Depression Rating Scale (HAM-D; [36]) and the Young Mania Rating Scale (YMRS; [37]). Lastly, the Global Assessment of Functioning (GAF) was performed on all patients. This assessment evaluates the individual’s psychological, social, and occupational functions on a scale from 0–100 with higher values representing a higher function in daily living [38].

### 2.3. Vitamin D

To assess the participants’ vitamin D status, we measured the 25(OH)D, which is composed of the sum of cholecalciferol (vitamin D_3_) and ergocalciferol (vitamin D_2_), and its principal catabolite 24,25(OH)_2_D, as well as calculating the vitamin D metabolite ratio (VMR). According to current recommendations from the Institute of Medicine (IOM), a 25(OH)D concentration < 50 nmol/L is deficient [39]. We used a recently published approach by Herrmann et al. (2023) to diagnose the functional vitamin D deficiency [19]. This approach requires a 24,25(OH)_2_D concentration < 3 nmol/L in combination with a VMR < 4%. If at least one of these criteria were fulfilled, the individuals were classified as having suboptimal vitamin D metabolism (see Table 1):

To determine vitamin D metabolites in serum samples of patients and healthy controls, we used a validated in-house liquid chromatography–tandem mass spectrometry (LC-MS/MS) method, which has been published previously. This spectrometry is able to simultaneously investigate 25(OH)D_3_, 25(OH)D_2_, 24,25(OH)_2_D_3_ and additionally 25,26(OH)_2_D_3_ with good selectivity, precision, and sensitivity. The inclusion of 24,25(OH)_2_D_3_ enabled us to define the vitamin D metabolism through the calculation of the VMR [40]. This method has performed satisfactorily in the vitamin D External Quality Assurance Scheme (DEQAS) for several years and is regularly controlled by internal quality control procedures.

### 2.4. Statistical Analyses

To assess differences in the vitamin D values between individuals with BD and healthy controls, we calculated an unpaired *t*-test. However, in case relevant assumptions for statistical t-tests were violated, Mann–Whitney *U*-Tests (MWU) were applied. Furthermore, to find possible correlations between vitamin D values and clinical parameters within the patients’ group, we implemented Spearman correlation analysis including bootstrapping. Hereby, a chi-square test (χ^2^) was used to determine distinctions in the functional vitamin D deficiency between patients and controls. Error probabilities below *p* < 0.05 were accepted to denote statistical significance and were not corrected for multiple comparisons due to the clinical setting of the study. IBM SPSS version 28 was used to perform data analyses.

## 3. Results

### 3.1. Descriptive Statistics

A total of 308 individuals, 170 BD patients and 138 healthy controls, were included in the statistical analyses. The two groups differed significantly in sex and age. Serum 25(OH)D was comparable in BD patients and healthy controls. Also, 24,25(OH)_2_D did not differ between the two groups. We also found no significant difference in VMR between individuals with BD and healthy controls (Table 2).

A chi-square test performed to compare the frequency of functional vitamin D deficiency between patients and controls showed no significant difference (see Table 3).

### 3.2. Vitamin D Status and Clinical Parameters

We found a significant inverse correlation between YMRS and 24,25(OH)_2_D and between YMRS and VMR. All other clinical parameters were unrelated to 25(OH)D, 24,25(OH)_2_D, and VMR (see Table 4).

## 4. Discussion

The aim of this investigation was to compare the vitamin D scores and vitamin D metabolism of individuals with BD and healthy controls. Furthermore, potential associations between markers of vitamin D metabolism and the clinical characteristics of BD were explored.

Contrary to our hypothesis, we did not find significant differences between BD patients and controls in the serum concentration of 25(OH)D, 24,25(OH)_2_D, or VMR. Interestingly, we found an inverse correlation between YMRS and 24,25(OH)_2_D, as well as with the VMR. The prevalence of functional vitamin D deficiency and sufficiency was comparable in the two groups.

Existing data related to vitamin D in BD is inconsistent; therefore, our results highlight important aspects regarding this topic. Previous studies described a proinflammatory status at the onset of BD and during the further course of the disease [41,42]. Vitamin D has an immunomodulatory activity, and thus may inhibit inflammatory processes in individuals at risk for BD [13]. Therefore, we would have expected a lower vitamin D metabolite concentration and an inferior functional vitamin D status in BD patients compared to healthy controls. However, our results did not support this hypothesis. The lack of significant differences may be explained by the fact that the present study was conducted in BD patients predominantly in a euthymic episode, with only subthreshold symptoms. Furthermore, the mean 25(OH)D concentration of the BD study group indicates that participants with BD were well-supplied with vitamin D. This is in line with some other studies also carried out on BD outpatients [43,44,45]. In contrast, studies exploring vitamin D status in inpatients with BD with severe and acute symptoms reported substantially lower levels of 25(OH)D [27,28,46]. Furthermore, a correlation between different phases of BD and vitamin D status has been reported in a recent review [21]. Nevertheless, there is limited evidence about acute manic episodes and status of vitamin D.

It has been speculated, that a decrease in vitamin D levels may contribute to an increase in intracellular Ca^2+^ concentration [47], leading to damage in the GABA-ergic system, resulting in manic symptoms. Altunsoy et al. (2018) reported a moderate inverse correlation between YMRS and low 25(OH)D concentration values [48]. They did not find a significant difference between BD patients in remission and healthy controls. Interestingly, Sikoglu et al. (2015) reported a reduction in manic symptoms measured by the YMRS in manic patients who were supplemented with vitamin D [45]. In line with these reports, we also found a negative correlation between YMRS and vitamin D, whereby our cohort included patients with hypomanic symptoms but not patients in an acute manic phase. In contrast to Altunsoy et al. (2018), the present study showed an inverse correlation between YMRS and 24,25(OH)_2_D, the main catabolite of vitamin D. Considering the fact that the correlation of 24,25(OH)_2_D with YMRS was obtained in euthymic or maximal hypomanic individuals in our cohort, it can be assumed that these markers may be helpful for assessing vitamin D status in the remission phases of the disease. However, studies that relate 24,25(OH)_2_D to clinical outcomes are still rare, and results should be interpreted cautiously [19]. Therefore, future studies should investigate the clinical relevance of this catabolite in mental disease.

In the existing body of literature, the exploration of vitamin D status was mainly carried out in samples with a heterogeneous psychiatric diagnosis, comparing vitamin D levels among psychiatric patients [21,26,27,28,48,49]. Belzeaux et al. (2015) reported more severe vitamin D deficiency in patients with mood disorders than in patients with schizophrenia [46]. On the contrary, Menkes et al. (2012) found more severe hypovitaminosis D in schizophrenic patients [27]. A recent literature review concluded that there is no difference in vitamin D status between BD patients and other psychiatric disorders [21]. Therefore, this inconsistency in the findings suggests that vitamin D deficiency could be a common feature of psychiatric patients, regardless of the psychiatric diagnosis. Further research in a longitudinal setting with good phenotypic data is needed to provide additional insights.

To date, all previous studies that analyzed vitamin D in mental diseases exclusively measured 25(OH)D. Through the additional determination of 24,25(OH)_2_D and VMR [17], the present study also analyzed vitamin D metabolism, and thus provides novel insights in the role of functional vitamin D deficiency in BD. Nevertheless, the prevalent frequency of functional vitamin D deficiency in our participants was rather low, with a prevalence of 9.6% in the BD patients’ group compared to 13.3% in controls. Consequently, the majority of participants were vitamin D-sufficient from a biochemical point of view, which might have influenced the outcomes of this study.

## 5. Limitations

Our study benefits from a substantial and diverse study population, encompassing a wide range of individuals suffering from BD. The sizable sample size, especially in comparison to other studies concerning the same topic [26,27,28,48,49], increases our analysis’s statistical power and enhances our results’ generalizability. To comprehensively explore the relationship between vitamin D and BD, we included and analyzed three different vitamin D values: 25(OH)D, 24,25(OH)_2_D_3,_ and the VMR. This approach allows for a more comprehensive understanding of the metabolic vitamin D functioning of the participants. Additionally, the vitamin D assessments were made using the validated LC-MS/MS, known for its precise and reliable quantification [18].

Our study assessed the clinical outcomes of the patient group by using several psychiatric tests, which on the one hand provide a detailed evaluation of the participants’ bipolar symptoms. On the other hand, the psychiatric assessments employed in this study provide a snapshot of the symptoms at the time of assessment and, therefore, may not fully capture the dynamic nature of the disease. Therefore, future research should incorporate more comprehensive, long-term parameters of BD to understand better the course of the disease, such as emotion recognition and cognitive and occupational functioning. Furthermore, the study did not assess the potential influence of medication intake on vitamin D levels or its impact on bipolar symptoms. Many individuals with BD are on medication that could affect both vitamin D metabolism and mood stability [50,51], making it an important consideration for further research. The intake of vitamin D supplements alone was an exclusion criterion. Apart from that, involving environmental aspects could be an interesting addition: for example, sun exposure and insufficiencies of the liver or kidney, which influences the in-body vitamin D production, or the intake of food products that have been proven to have a high vitamin D content [52]. By checking patients at a wide variety of testing times over the course of several years, we tried to simulate the variability of vitamin D levels depending on the season. Also, the season in which the blood sample was taken could be a meaningful covariable in future research. More specific questionnaires could help in the future. Moreover, our study did not differentiate between genders in the analysis. Gender-based differences in vitamin D metabolism and susceptibility to BD may exist and could also enrich research [53,54].

Our sample represents predominantly euthymic patients, so our ability to explore the relationship between vitamin D and mood fluctuations is limited. Further exploration of vitamin D status in different phases of the disease may provide additional insights.

Despite these limitations, our study contributes to the growing body of knowledge surrounding vitamin D and BD. Further research should aim to address these gaps, to provide a more nuanced understanding of the complex interplay between these two topics.

## 6. Conclusions

In summary, BD patients appear to have a comparable vitamin D status to healthy controls, according to our results. Moreover, based on the inverse correlation of 24,25(OH)_2_D and VMR with YMRS, we hypothesize that hypomanic symptoms might display higher energy, and often an agitation, in hypomanic episodes which lead to the higher turnover of vitamin D in the body or lower nutritional intake. Based on the small effect size and the predominantly euthymic sample in our study, further exploration in individuals with manic symptoms would be needed to confirm this association. In addition, the psychometric tests at one time-point used in this study represent only a snapshot that may vary over time. Therefore, long-term clinical markers and an assessment in different phases of the disease may provide valuable additional insights.

## Figures and Tables

**Table 1:** Classification of groups based on functional information on vitamin D metabolism.

Functional vitamin D deficiency	Both criteria fulfilled	VMR ≤ 4% and 24,25(OH)D_2_ ≤ 3 nmol/L
Suboptimal vitamin D metabolism	One criterium fulfilled	or
Functional vitamin D sufficiency	No criterium fulfilled	VMR ≥ 4% and 24,25(OH)_2_D ≥ 3 nmol/L

Note: Criteria 1 is VMR ≤ 4%; criteria 2 is 24,25(OH)_2_D ≤ 3 nmol/L.

**Table 2:** Patients’ and controls’ characteristics.

	Patients (*n* = 170)	Controls (*n* = 138)	Statistics
Sex			*χ*^2^ (1) = 9.260, *p* = 0.002 *
Female	84 (49.4%)	92 (66.7%)	
Male	86 (50.6%)	46 (33.3%)	
Age, Mean ± SD (IQR)			
	43.9 ± 12.8 (32.9–52.8)	36.5 ± 14.8 (24.2–50.9)	*U* = 7845.000, *Z* = −4.999, *p* < 0.001 *
BMI (kg/m^2^), Mean ± SD (IQR)			
	27.8 ± 5.9 (23.8–30.2)	24.1 ± 4.5 (21.1–26.6)	*U* = 4441.000, *Z* = −5.716, *p* < 0.001 *
25(OH)D (nmol/L), Mean ± SD (IQR)			
	57.8 ± 24.3 (39.5–72.9)	61.5 ± 29.3 (42–72.2)	*U* = 10,956.000, *Z* =–.996, *p* = 0.319
< 30	20 (11.8%)	13 (9.4%)	*U* = 129.000, *Z* = −0.037*, p* = 0.971
30–50	44 (25.9%)	32 (23.2%)	*t (74)* = 0.054, *p* = 0.957
> 50	106 (62.4%)	93 (67.4%)	*U* = 4778.500, *Z* = −0.371, *p* = 0.710
24,25(OH)_2_D (nmol/L), Mean ± SD (IQR)			
	3.8 ± 2.4 (2–5.2)	4.2 ± 2.7 (2.4–5.5)	*U* = 10,573.000, *Z* = −1.489, *p* = 0.137
<3	67 (39.4%)	43 (31.2%)	*U* = 1396.500, *Z* = −0.270, *p* = 0.788
>3	103 (60.6%)	95 (68.8%)	*U* = 4659.500, *Z* = −0.578, *p* = 0.563
VMR (%), Mean ± SD (IQR)			
	6.4 ± 2.2 (4.9–8.2)	6.8 ± 2.4 (5.3–8.4)	*t* (306) = −1.601, *p* = 0.110
<4	23 (13.5%)	17 (12.3%)	*U* = 165.500, *Z* = −0.821, *p* = 0.412
>4	147 (86.5%)	121 (87.7%)	*U* = 7910.500, *Z* = −1.557, *p* = 0.120

Note: SD = Standard Deviation; IQR = interquartile range; BMI = body mass index; VMR = vitamin D metabolite ratio; SD = standard deviation; * Significance set at *p* < 0.05.

**Table 3:** Vitamin D status regarding the functional information on vitamin D metabolism.

	Patients (*n* = 170)	Controls (*n* = 138)	
Deficient	23 (13.5%)	17 (12.3%)	*χ*^2^ (2) = 2.555, *p* =.279
Suboptimal	44 (25.9%)	26 (18.8%)	
Sufficient	103 (60.6%)	95 (68.8%)	

**Table 4:** Correlation of vitamin D values with clinical parameters in patient group.

	Mean ±SD (IQR)	25(OH)D	24,25(OH)2D	VMR
SA	0.4 ± 0.69 (0–1)	r = 0.023, *p* = 0.819	r = −0.004, *p* = 0.968	r = −0.042, *p* = 0.671
BDI	18.4 ± 12 (8–29)	r = 0.073, *p* = 0.462	r = 0.034, *p* = 0.730	r = −0.098, *p* = 0.322
HAM-D	7.1 ± 5.8 (3–10.8)	r = 0.040, *p* = 0.684	r = 0.030, *p* = 0.765	r = −0.005, *p* = 0.957
YMRS	1.7 ± 3.6 (0–1)	r = −0.127, *p* = 0.197	r = −0.220, *p* = 0.025 **	r = −0.238, *p* = 0.015 **
GAF	65.9 ± 13.1 (56.5–70)	r = −0.011, *p* = 0.915	r = 0.031, *p* = 0.772	r = 0.114, *p* = 0.287

Note: *n* = 104; SD = Standard Deviation; IQR = interquartile range; SA = suicide attempts; BDI = Beck Depression Inventory; HAM-D = Hamilton Depression Rating Scale; YMRS = Young Mania Rating Scale; GAF = Global Assessment of Functioning; VMR = Vitamin D metabolite ratio ** Significance set at *p* < 0.05.

## Data Availability

Data are contained within the article.
